# Inlay osteotome sinus floor elevation with concentrated growth factor application and simultaneous short implant placement in severely atrophic maxilla

**DOI:** 10.1038/srep27348

**Published:** 2016-06-02

**Authors:** Yonghui Chen, Zhiyu Cai, Dingguo Zheng, Pei Lin, Yahua Cai, Shuxin Hong, Yiwei Lai, Dong Wu

**Affiliations:** 1 Department of Stomatology, Zhangzhou Affiliated Hospital of Fujian Medical University, Zhangzhou 363000, China; 2 School and Hospital of Stomatology, Fujian Medical University, Fuzhou 350002, China; 3 Department of Stomatology, Fujian Medical University Union Hospital, Fuzhou 350001, China

## Abstract

Sinus floor elevation with simultaneous implant placement in severely atrophic maxilla is challenging. The aim of this retrospective study was to evaluate the short-term performance of modified osteotome sinus floor elevation (OSFE) with concentrated growth factor (CGF) application and concurrent placement of a short implant in cases with residual bone height (RBH) of 2–4 mm. Twenty-five short implants were installed in 16 patients with mean RBH of 3.23 mm using modified OSFE with CGFs from January 2012 to April 2014. Postoperatively, the implants were clinically evaluated, and vertical bone gain (VBG) was measured using cone beam computed tomography. The mean duration of follow-up was 19.88 months (12–32 months). All the implants were stable with an overall survival rate of 100%. The mean VBG immediately after surgery was 9.21 mm. Six months later, significant reduction of alveolar bone height (2.90 ± 0.22 mm) was found (P < 0.05). During the second 6-month period, further alveolar bone resorption (0.14 ± 0.11 mm) was noted but without significance (P > 0.05). Within the limits of this study, modified OSFE with CGF application and simultaneous short implant placement could yield predictable clinical results for severely atrophic maxilla with RBH of 2–4 mm.

Teeth missing from the posterior maxilla for long periods of time always ensure sinus pneumatization and edentulous alveolar ridge resorption, leading to insufficiency of the residual alveolar bone height (RBH) for implant-supported rehabilitation. To solve this problem, various surgical techniques have been proposed. Among them, the most commonly adopted strategy for bone augmentation at the sinus floor is the lateral sinus floor elevation (LSFE) technique. Initially introduced by Boyne, this surgical procedure achieves exposure of the maxillary sinus cavity through a bony window created in its front wall, followed by Schneiderian membrane lifting and bone grafting^1^. Although this technique has a predictable success rate, it is time-consuming and traumatic. For minimally invasive purposes, in 1994, Summers introduced the osteotome sinus floor elevation (OSFE) technique^2^. Unlike LSFE, this technique affords direct access to the sinus via the alveolar crest. After the sinus floor at the implant site is fractured and apically elevated together with its overlying sinus membrane by osteotomes with increasing diameters, grafting material is packed into the newly created, confined space. Compared with LSFE, OSFE reduces the extent of the surgical site, preserves the bone block at the access with its vascularization, and condenses the adjacent residual bone using a progressively sized series of osteotomes. These advantages could contribute to the reduction in surgical time, attenuation of postoperative discomfort, enhancement of implant primary stability, and promotion of osseointegration. According to the literature, the OSFE technique yields comparable results to LSFE^3^,^4^. OSFE has been documented to be a reliable surgical procedure in cases with RBH between 6 and 8 mm^5^. Recently, the indications for OSFE were successfully extended to cases of atrophic maxillae in which the RBH ranges from 4 to 6 mm^6^,^7^. For cases with RBH no greater than 4 mm, a few authors have reported successful results of OSFE^8^,^9^,^10^. In a multicentre study to compare the performance of implant placement using the OSFE technique in patients with RBH ≤4 mm and RBH >4 mm, no significant difference was observed in terms of success rate or peri-implant bone loss^10^. In another prospective, randomized, controlled study^8^,^9^, implants were placed by OSFE in patients with an average RBH of 2.4 ± 0.9 mm. The success rate was 91.9% after 3 years, which was comparable to that of implants placed with the LSFE technique^11^. In contrast, other studies have shown that severely atrophic alveoli reduced the implant survival rate^12^,^13^. In a multicentre, retrospective study, Rosen PS *et al*. found that the survival rate of implants placed using the OSFE technique in cases with RBH no greater than 4 mm was 85.7%, which was significantly less than that in cases with RBH no less than 5 mm^12^. Toffler M also reported that the survival rate of implants was only 73.3% in cases with RBH of 4 mm or less^13^. Lack of primary stability, limitations in the extent of sinus membrane elevation and risk of membrane perforation have restricted the application of OSFE in cases with RBH less than 4 mm^14^. However, because the prevalence of severely atrophic maxilla is high^15^, it is of great clinical interest to investigate viable techniques for sinus floor elevation and immediate implant placement in cases of edentulous posterior maxillae with RBH <4 mm.

In cases of severely maxillary resorption, conventional implants might be too long to be placed into the augmented sinus floor. Implants with reduced length are considered to be an appropriate alternative. With improvement in implant surface modifications, the past decade witnessed a paradigm shift in crown-implant ratio for the long-term success of implants. A meta-analysis conducted in 2014 revealed similar success rates of short implants to those of implants with conventional lengths. This study also showed the length of implants not to be a critical factor determining implant success^16^. Although a retrospective cohort study reported that short implants 6 mm in length in the maxillary posterior area could only achieve a survival rate of 87%^17^, most other long-term studies with up to 10 years of follow-up have shown that short implants in the posterior maxilla could obtain satisfying clinical outcomes^18^,^19^. In a recently published systematic review, short implants in the augmented sinus were documented to be comparable to conventional implants in terms of clinical and radiographic outcomes. Furthermore, short dental implants could reduce patient discomfort, morbidity, costs, surgical time and biological complications as well^20^.

Platelet concentrates have been shown to be a promising scaffold in tissue regeneration. Platelet concentrates are autologous, easy to prepare at the chairside, and full of high concentrations of growth factors^12^. *In vitro* studies have proved the effects of these signalling molecules on cell proliferation, migration, differentiation and matrix synthesis^21^. In recent years, platelet concentrates have been applied in sinus floor elevation and bone grafting. Platelet-rich plasma (PRP), which was among the first generation of platelet concentrates^22^, has been used with autogenous bone or bone substitute in sinus augmentation but without significantly positive effects^23^,^24^,^25^. Platelet-rich fibrin (PRF) is from the second generation of platelet concentrate products. PRF has many advantages over PRP, including osteogenic ability, a simple preparation process, absence of extrinsic biological agents, and sustained release of growth factors^26^. Previous studies have revealed the potential of PRF to promote endosinus bone regeneration^27^,^28^ and to reduce healing time after sinus floor elevation^26^. Developed by Sacco in 2006, concentrated growth factors (CGFs) are produced by a centrifuge device (Medifuge Silgradent srl, Italy) in a similar manner to PRF but with different speeds. Compared with PRF, the fibrin matrix of CGFs is larger, denser and richer in growth factors. Therefore, CGFs could be expected to have better properties for clinical manipulation and regenerative potential. It has been reported that application of CGFs could significantly increase bone formation in the construction of bone defects^29^. CGFs have also demonstrated their potential in accelerating osteogenesis associated with guided bone regeneration in sinus augmentation^30^.

Owing to the minimal invasiveness of the OSFE technique, the reliability of short implants and the regenerative potential of CGFs, clinicians are using these techniques in combination to treat patients with advanced maxillary atrophy. However, to date, there have been no reports of the performance of OSFE using CGFs as the grafting material with immediate short implant placement in cases with RBH between 2 and 4 mm. The aim of this retrospective study was to investigate whether the proposed protocol could be a feasible therapy strategy for severely atrophic maxillae with RBHs ranging from 2 to 4 mm.

## Methods

### Study Population and Design

This was a retrospective observational study of 25 tapered short implants (4.5–7.0 mm in diameter and 7–8 mm in length) (Dentium, Seoul, South Korea) placed simultaneously with OSFE using CGFs in 16 patients (10 males and 6 females) (ages ranging from 21 to 68 years, mean 54.2 ± 10.4 years) between January 2012 and April 2014. The patients selected for this study were treated with the proposed protocol in the Department of Stomatology, Affiliated Zhangzhou Hospital of Fujian Medical University. The patients were informed about the procedures for the study. Written consent was obtained. The study design and clinical procedures were performed in accordance with the Helsinki Declaration (revised in 2008) and were approved by the Ethics Committee of Affiliated Zhangzhou Hospital of Fujian Medical University.

**Figure 1:**
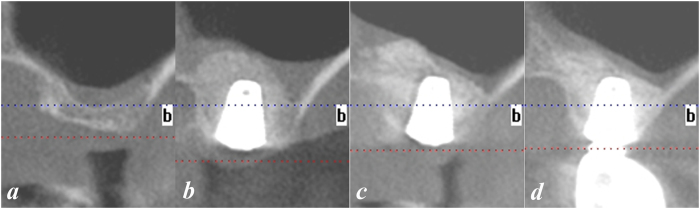
Radiographic measurements. CT scans were obtained before (**a**), immediately after (**b**), at 6 months (**c**) and at 1 year (**d**) after implant placement.

Patients were included in this study on the basis of the following criteria:

1. at least 18 years of age;
2. missing a maxillary molar for more than 5 months;
3. adequate oral hygiene, that is, full-mouth bleeding score and plaque score less than 25% at baseline;
4. adequate vertical occlusal dimensions for the restoration of an anatomically formed crown;
5. bone height beneath the maxillary sinus between 2 and 4 mm on panoramic radiography and cone beam CT (CBCT) imaging (Fig. 1a);
6. an adequate residual alveolar ridge width for implant placement;
7. absence of maxillary sinus pathology radiographically (thickness of the sinus membrane no greater than 2 mm) and clinically; and
8. absence of maxillary sinus septa on CBCT.

### CGF preparation

**Figure 2:**
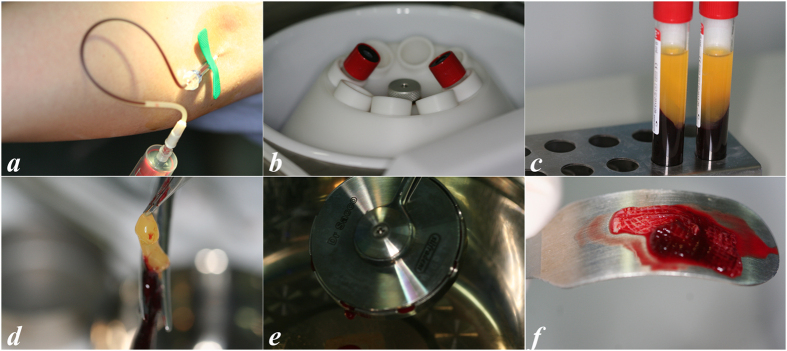
Preparation of CGFs. Blood collection (**a**); blood centrifugation (**b**); after centrifugation (**c**); removal of the red blood cell layer (**d**); CGF clot compression (**e**); CGF membrane (**f**).

CGF was prepared as described by Bozkurt *et al*.^31^. Immediately before surgery, 30–50 ml (3–5 tubes) of whole blood were drawn into 10-ml glass-coated plastic tubes without anticoagulant reagent and were centrifuged with a device (Medifuge, Silfradenstsr, S. Sofia, Italy) using the following built-in programme: 30” acceleration, 2′ 2700 r.p.m., 4′ 2400 r.p.m., 4′ 2700 r.p.m., 3′ 3000 r.p.m., and 36” deceleration and stop. The total spin time was approximately 14 minutes. The whole blood was divided into four layers: (1) the bottom red blood cell layer; (2) the second growth factor and stem cell layer (CGF); (3) the third buffy coat layer; and (4) the top serum layer. The CGF layer was separated using sterile scissors. CGF clots were pressed into membranous film with a constant thickness of 1 mm (Fig. 2).

### Surgical procedure

**Figure 3:**
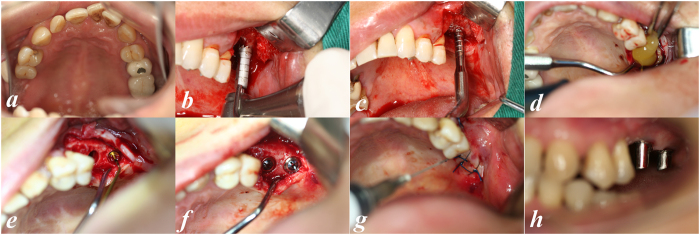
Modified inlay OSFE with CGF application and concurrent implant placement. Edentulous alveolar ridge in the left maxilla before surgery (in mirror) (**a**); trephine was advanced to a depth 0.5 to 1 mm away from the sinus floor (**b**); CGF was packed into the space under the sinus membrane (**c**); the alveolar bone core was then fractured and displaced apically with an osteotome (**d**); The Schneiderian membrane was elevated with proprietary sinus membrane elevators (**e**); the cover screw was placed (**f**); after suturing (**g**); abutment placement 6 months later.

All the surgeries were performed by the same oral surgeon with more than 15 years of experience. One hour before surgery, the patient received 2 g of amoxicillin and clavulanic acid (Augmentin, SmithKline Beecham Pharmaceuticals, UK) as a prophylactic regimen. Before anaesthesia, the patient rinsed with 0.12% chlorhexidine gluconate for 1 minute. Local anaesthesia was achieved with articaine chlorhydrate 4% and adrenaline 1:100.000 (Primacaine, Pierre Rolland, France). A full-thickness midcrestal incision was made, and vertical releasing incision was made if necessary. The alveolar ridge was exposed by buccal and palatal flap reflection. The flap elevation was minimized but sufficient to provide adequate access. The implant position was marked on the alveolar crest with a small round drill (Ø 2.0 mm). Subsequently, an internally irrigated trephine (two sizes smaller than the implant) drill was advanced to a depth 0.5 to 1 mm away from the sinus floor, as measured from the preoperative CBCT. After removal of the trephine burr, the bone core was left on the alveolar ridge. Then, a calibrated osteotome was chosen corresponding to the diameter of the trephine in preparation. The alveolar bone core was then fractured and displaced apically by gently tapping it with malleting force. In this manner, the alveolar bone core with overlying Schneiderian membrane was elevated. Special attention was paid to avoiding sinus membrane perforation. The integrity of the Schneiderian membrane was assessed by the Valsalva manoeuvre^8^ and manually with a depth gauge. Proprietary sinus membrane elevators (Dentum, South Korea) were then used circumferentially to detach the Schneiderian membrane from the sinus floor. CGF gel and then membrane were packed into the space below the elevated Schneiderian membrane. Through the elevation manoeuvre and compression of CGFs into the implant site, the Schneiderian membrane was smoothly separated from sinus floor until the adequate vertical height was obtained. The site was then prepared with the appropriate osteotomes to one size smaller than the selected implant. In cases without perforation of the Schneiderian membrane, Bio-Oss bone substitute was packed into the obtained space beneath the CGF membrane. In the cases with sinus membrane perforation, no bone substitute was used. The implant was installed with an insertion torque of 30 Ncm, and the cover screw was placed. Finally, the flap was repositioned and sutured (Fig. 3). Patients underwent CBCT examination immediately after surgery (Fig. 1b).

### Postsurgical Treatment

After surgery, all the patients received oral antibiotics as prophylactic measure for an additional 3–5 days and nonsteroidal analgesics for 3–5 days. The patients were given oral hygiene instructions including: (1) mouth rinsing with 0.12% chlorhexidine for 2 weeks; (2) avoidance of tooth-brushing around the implant site for 7 days; and sinus-specific instructions including: (1) no sipping through a straw; (2) avoidance of blowing; (3) sneezing with an open mouth; and (4) antihistamine medication for 72 hours. The sutures were removed at 2 weeks postoperatively. The site was allowed to heal for 6 months. Then, a healing abutment was placed, followed by temporary restoration. Permanent restoration was completed 3 months later.

### Postoperative Examination and Data Collection

Follow-up recalls were scheduled for 2 weeks and for 1, 3, 6 and 12 months during the first year and annually thereafter. Clinical examination was conducted at each visit. Variables referred to the method by Tachieri *et al*.^13^ and included the following:

1. success of the prosthesis, evaluated by function and stability;
2. implant success according to conventional criteria^10^; and
3. patients’ satisfaction with the rehabilitation of mastication, phonetics, and anaesthetics, assessed by questionnaires using a five-point scale, ranging from 0 (fully unsatisfied) to 4 (fully satisfied) for each question^11^.

Patients underwent a CBCT scan at the 6- and 12-month recalls (Fig. 1c,d). Radiographic measurements referred to the method by Teng *et al*.^9^. The RBH value was calculated according to the measurement on CBCT images: RBH = 1/3(M + C + D) (M: height of the mesial alveolar residual crest, C: height of the central alveolar residual crest, D: height of the distal alveolar residual crest). The alveolar bone height immediately after surgery (ASBH), the bone height before prosthesis restoration at 6 months postoperatively (BRBH) and that at the 12-month revisit (RVBH) were measured and calculated in the same fashion. The vertical bone gain (VBG) of the implant site was the difference between ASBH and RBH (VBG = ASBH-RBH). The vertical bone resorption over the first 6 months (VBR1) was the difference between BRBH and ASBH (VBR1 = ASBH-BRBH). The vertical bone resorption over the second 6 months (VBR1) was the difference between RVBH and BRBH (VBR2 = BRBH-RVBH).

Follow-up clinical examinations and radiographic measurements were performed by two dentists who were not involved in the surgical procedures. Any disagreement was resolved by choosing the less favourable result.

### Statistical Analysis

Data regarding alveolar bone height gain at different time points are presented as the mean values ± standard deviations (x ± SD) and were compared with each other using the paired t-test. Survival rates for implants were calculated by Kaplan-Meyer analysis. Statistical analysis was performed with SPSS software (Statistics software v 19.0; IBV Corp, Armonk, NY). P values < 0.05 were considered statistically significant.

## Results

In our study, we evaluated 18 sinus floor augmentations accomplished with immediate placement of 25 implants into edentulous molar sites (15 implants at the first molar site and 10 implants at the second molar site) (Table 1). Two sinus membrane perforations were detected for an overall perforation rate of 11.1%. These two perforations were discovered after sinus floor membrane elevation. All the implants were clinically stable without pain or other complications. All the prosthetic rehabilitations were successful. The survival rate of the implants was 100% during the observation period of 19.88 ± 4.80 months. The mean RBH of the alveolar crest was 3.23 ± 0.39 mm (range, 2–4 mm). Immediately after surgery, the mean alveolar bone height was 12.44 ± 0.33 mm, with vertical bone gain at 9.21 ± 0.66 mm. Six months postoperatively, significant resorption of the alveolar bone could be detected by CBCT imaging evaluation. The mean alveolar bone height at the 6-month recall was 9.54 ± 0.48 mm. A significant reduction of alveolar bone height (2.90 ± 0.22 mm) was observed during the first 6 months after surgery (P < 0.05). Twelve months after the operation, the mean alveolar bone height was 9.40 ± 0.47 mm. During the second 6-month period, further alveolar bone resorption (0.14 ± 0.11 mm) could be noted by CBCT measurement but without significance (P > 0.05). All the patients returned their questionnaires, and all of them reported full satisfaction regarding function, phonetics, and aesthetics.

## Discussion

Our study found a 100% survival rate of implants and satisfying alveolar bone gain within the observation period, indicating that the proposed protocol could achieve successful results in patients with severely atrophic maxillae (2 mm <RBH <4 mm). To the best of our knowledge, this was the first retrospective study that evaluated the clinical effects of OSFE using CGFs as the grafting material and simultaneous short implant placement for atrophic maxillae with RBH less than 4 mm.

In agreement with the results of previous studies of the performance of short implants in simultaneous sinus elevation^18^,^19^, our data also demonstrated a high survival rate of implants with reduced length. Long-term success of implant rehabilitation necessitates the primary stability of the implant in the early phase and implant-bone integration through bone regeneration and remodelling in the late phase. Achieving primary stability is especially challenging in cases with severely atrophic posterior maxillae. Efforts have been undertaken to increase the primary stability of implants by clinicians and manufacturers. Primary stability could be enhanced by modifying the surgical technique for implant placement. Studies have shown that the undersized technique, in which a final drill with a diameter smaller than the implant diameter, obtains superior implant stability to the press-fit technique^32^. Compared with the bone-drilling technique, bone-condensing techniques have also been reported to result in greater primary stability^33^. Implant design is another factor in increased primary stability. It has been reported that implants with tapered shapes^34^, deep threads^35^, and surface roughness^36^,^37^ could achieve greater primary stability. In addition, because the implant surface area is proportional to the square of its diameter, wide diameter implants could dramatically increase bone-implant contact, thus reinforcing primary stability. In our study, in order to achieve primary implant stability in alveolar bone with RBH less than 4 mm, an osteotome bone-condensing technique was used. Moreover, a taper-shaped, deep dual thread, SLA (sand-blasted, large-grit, acid-etched) rough surface Superline implant of reduced length (7–8 mm) and wide diameter (4.5 to 7.0 mm) was chosen to increase mechanical support from the available surrounding bone.

In the traditional OSFE technique, before the sinus floor is fractured and displaced apically, alveolar bone is removed with twist drills or burrs to prepare a channel for the placement of osteotomes. In our study, an modified inlay OSFE was performed^9^. Unlike conventional OSFE, a trephine was used instead of a drill to prepare a bone core at the recipient site in the inlay OSFE. The advantages of this technique include the following: (1) minimal invasiveness and time savings due to the elimination of repeated drilling and hammering; (2) less usage of bone substitute and cost-efficiency owing to maximal preservation of alveolar bone at the site of implant placement; (3) stable space maintenance because of the tent-peg effect of the apically displaced vascularized bone core; and (4) better osteogenesis compatibility owing to the osteoinductive properties of the autogenous bone core^38^.

Our results showed remarkable vertical bone augmentation (9.21 ± 0.66 mm immediately after surgery) with the proposed protocol. The intended vertical dimension gain of alveolar bone in sinus elevation is largely determined by the RBH before surgery and the length of the implant. For cases with RBH greater than 6 mm, most previous studies have reported an average bone height gain of 3–4 mm using either the LSFE or the OSFE approach,^13^,^39^,^40^ although greater degrees of elevation have been attainable^41^,^42^. In contrast, much greater bone height augmentation has been achieved in cases of severely atrophic maxillae. The LSFE approach allows for an increase of more than 10 mm in bone height^43^. Some studies employing the OSFE technique have also achieved remarkable vertical dimension elevation in patients with severely resorbed ridges with RBH <4 mm^8^,^9^,^44^. Nedir R *et al*. placed thirty-seven 8 mm-long implants in 12 patients with a mean maxillary RBH of 2.4 ± 0.9 mm, using an OSFE procedure with or without grafting. After a 3-year follow-up, the endosinus bone gain of the implants in the grafting group was 5.1 ± 1.2 mm, whereas that in the non-grafting group was 4.1 ± 1.0 mm^9^. In a 60-month clinical and radiological study^44^, Lo Giudice G *et al*. evaluated the performance of the OSFE technique in cases with RBH less than 3 mm. Forty-five implants 8.5–11 mm in length were inserted in 31 patients with an average RBH of 2.0 ± 0.5 mm. The results of their study showed an excellent survival rate (99.5%) and considerable bone height gain (7.8 ± 0.86 mm). A recent study using an inlay osteotome procedure similar to ours but with shorter implants (5.7–6.0 mm) reported 5.38 mm as the mean bone height gain^9^. In the present study, because of the associated use of implants 7–8 mm in length, greater sinus floor lifting is needed. To achieve this objective, strict inclusion criteria, usage of grafting material with superior properties, and optimization of surgical manoeuvres have been adopted. Firstly, to avoid restriction and perforation during sinus membrane elevation, patients with sinus septa were excluded by radiographic examination. Secondly, CGF membrane beneath the bone core and sinus membrane might serve as a cushion during sinus lifting to dampen the compressive force by the osteotome. Thirdly, combined usage of the osteotome and sinus membrane elevators could contribute to maximum detachment of the Schneiderian membrane from the sinus floor. Finally, the bone core located apical to the implant may have played a critical role in maintaining the Schneiderian membrane at a high position. During the first 6 months, the augmented bone experienced significant resorption, particularly the bone core apical to the implant. The reduction in the alveolar bone height was 2.90 ± 0.22 mm. Other authors using the inlay osteotome protocol also reported obvious resorption of the bone core^9^. Compared with the relatively stable vertical bone height after surgery using the traditional osteotome technique^13^, it seems that resorption would occur in the apically displaced bone core during early stages postoperatively. Nevertheless, the bone core might have acted as a “placeholder” to provide the necessary space, facilitating bone generation around the implant as observed radiographically at the 6-month recall. During the second 6 months, only insignificant alveolar bone resorption was noted, indicating less dynamic bone remodelling during the late stage after surgery.

Certainly, greater bone height augmentation will increase the incidence of membrane perforation. In the present study, sinus membrane perforation was detected in 2 cases. The invisibility of the sinus floor has been considered to be the major disadvantage of OSFE^5^,^31^. However, compared with LSFE, a lower incidence of intrasurgical complications, including sinus membrane perforation, was reported using the OSFE approach^45^. In addition, a previous study showed that the occurrence of perforation was not correlated with the success rate of implants, even without grafting material^46^. In our study, to avoid displacement of bone substitute into the sinus cavity, CGF membrane was used as the only grafting material in the two cases of sinus membrane perforation. CGF was packed into the site of membrane perforation as a ceiling for the obtained dome-shaped space. Undetectable perforation might also occur in other cases because of the extensive membrane elevation. In any case, the adhesive property and regenerative capacity of CGFs could contribute to obliteration of the perforation site and soft tissue healing^47^. The study of Toffler *et al*. demonstrated a high success rate with OSFE using only PRF at sites with RBH ranging from 5 to 8 mm. Because CGFs have better biological and physical properties, it would be interesting to investigate the performance of CGFs as the only grafting material in sinus augmentation with RBH less than 5 mm in future studies.

Our present research focused on the performance of the modified OSFE technique in cases of severely atrophic alveoli, and strict inclusion criteria for the patients were applied, including the absence of sinusitis. Because mucosal thickening of more than 2 mm has been reported to indicate sinusitis^48^,^49^, the sinus membrane thickness in all our patients was no more than 2 mm. As a consequence, the data we collected were not very appropriate for determining the correlation between RBH and sinus membrane thickness. Although this information is not within the scope of the present study, it is very interesting and deserves further investigation.

Despite the strict anatomical inclusion criteria and the skill demanded for the operation in the present study, the results indicated that the protocol presented could allow for simultaneous sinus floor elevation and implant installation in atrophic posterior maxillae with RBH between 2 mm and 4 mm, extending the indications for implant rehabilitation. Studies with randomized designs, larger sample sizes and long-term follow-up are needed to validate this protocol.

## Additional Information

**How to cite this article**: Chen, Y. *et al*. Inlay osteotome sinus floor elevation with concentrated growth factor application and simultaneous short implant placement in severely atrophic maxilla. *Sci. Rep.* **6**, 27348; doi: 10.1038/srep27348 (2016).

## Figures and Tables

**Table 1:** Variable and Results of 25 implants during the Study Period.

**No.**	**Sex**	**Age (Years)**	**Site**	**Implant**		**Before Surgery**	**After Surgery**		**6 months after Surgery**		**12 months after Surgery**		**Last follow-up after surgery (month)**
				**D (mm)**	**L (mm)**	**RBH (mm)**	**ASBH (mm)**	**VBG (mm)**	**BRBH (mm)**	**VBR1 (mm)**	**RVBH (mm)**	**VBR2 (mm)**	
1	M	68	#3	6	8	3.42	12.24	8.82	9.14	3.1	9.12	0.02	32
2	M	56	#2	6	8	3.18	12.61	9.43	9.72	2.89	9.56	0.16	18
			#14	6	7	2.35	12.69	10.34	9.86	2.83	9.68	0.18	18
			#15	5	8	3.56	12.36	8.8	9.21	3.15	9.18	0.03	18
3	F	52	#14	5	8	3.29	12.35	9.06	9.25	3.1	9.19	0.06	24
4	M	21	#14	6	8	3.35	12.18	8.83	9.12	3.06	9.07	0.05	12
5	M	49	#2	5	8	3.69	12.09	8.4	9.08	3.01	8.96	0.12	24
6	F	60	#14	5	8	3.09	12.29	9.2	9.19	3.1	9.17	0.02	18
			#15	4	8	2.67	12.50	9.83	9.72	2.78	9.52	0.2	18
7	M	62	#14	5	8	3.43	11.96	8.53	8.92	3.04	8.61	0.31	15
8	M	55	#3	5	7	2.31	12.98	10.67	10.21	2.77	9.95	0.26	24
			#2	6	8	3.12	12.45	9.33	9.66	2.79	9.49	0.17	24
9	M	49	#3	7	8	2.98	12.75	9.77	9.78	2.97	9.56	0.22	16
10	M	48	#14	5	8	3.32	12.49	9.17	9.71	2.78	9.59	0.12	15
			#15	5	8	3.41	12.18	8.77	9.23	2.95	9.19	0.04	24
11	M	58	#3	6	8	3.59	11.98	8.39	9.14	2.84	9.11	0.03	18
12	F	53	#3	5	8	3.12	12.85	9.73	9.97	2.88	9.85	0.12	20
			#2	5	8	3.46	12.32	8.86	9.56	2.76	9.41	0.15	19
13	F	61	#3	6	8	3.55	12.29	8.74	9.57	2.72	9.55	0.02	30
14	M	57	#14	5	8	3.64	11.99	8.35	9.15	2.84	9.11	0.04	24
15	F	55	#14	6	8	3.18	12.89	9.71	10.15	2.74	10.04	0.11	18
			#15	5	8	2.88	13.01	10.13	10.61	2.4	10.19	0.42	15
			#3	5	8	3.31	12.51	9.2	9.81	2.7	9.76	0.05	20
			#2	5	8	2.99	12.96	9.97	10.25	2.71	10.16	0.09	15
16	F	63	#15	6	8	3.89	12.16	8.27	8.56	3.6	8.16	0.4	18
***Total Average***		***54.2***				***3.23***	***12.44***	***9.21***	***9.54***	***2.90***	***9.40***	***0.13***	***19.88***

Variables and results of 25 implants in 16 patients during the observation period. M, male; F, female; D, diameter; L, length; RBH, residual bone height before surgery; ASBH, bone height after surgery; VBG, vertical bone gain after surgery; BRBH, bone height before the restoration at 6-month recall; VBR1, vertical bone resorption during the first 6 months; RVBH, bone height at 12-month recall; VBR2, vertical bone resorption during the second 6 months.
